# Perceived Social and Built Environment Correlates of Transportation and Recreation-Only Bicycling Among Adults

**DOI:** 10.5888/pcd15.180060

**Published:** 2018-11-08

**Authors:** Anna K. Porter, Harold W. Kohl, Adriana Pérez, Belinda Reininger, Kelley Pettee Gabriel, Deborah Salvo

**Affiliations:** 1 Department of Epidemiology, Human Genetics, and Environmental Sciences, The University of Texas Health Science Center at Houston School of Public Health in Austin, Austin, Texas. Dr. Porter is now with the Department of Epidemiology, The University of North Carolina at Chapel Hill Gillings School of Global Public Health, Chapel Hill, North Carolina; 2 Department of Epidemiology, Human Genetics, and Environmental Sciences, The University of Texas Health Science Center at Houston School of Public Health in Austin, Austin, Texas; Department of Kinesiology and Health Education, University of Texas at Austin College of Education, Austin, Texas; 3 Department of Biostatistics and Data Science, The University of Texas Health Science Center at Houston School of Public Health in Austin, Austin, Texas; 4 Department of Health Promotion and Behavioral Sciences, The University of Texas Health Science Center at Houston School of Public Health in Brownsville, Brownsville, Texas; 5 Department of Epidemiology, Human Genetics, and Environmental Sciences, The University of Texas Health Science Center at Houston School of Public Health in Austin, Austin, Texas, and The University of Texas at Austin, Dell Medical School, Department of Women’s Health, Austin, Texas; 6 Department of Epidemiology, Human Genetics, and Environmental Sciences, The University of Texas Health Science Center at Houston School of Public Health in Austin, Austin, Texas, and Center for Nutrition and Health Science Research, National Institute of Public Health of Mexico, Cuernavaca, Morelos, Mexico. Dr Salvo is now with the Brown School, Prevention Research Center in St Louis, and Center for Diabetes Translation Research, Washington University in St Louis, St Louis, Missouri

## Abstract

**Introduction:**

Research on perceptions of environmental factors in relation to transportation and recreation bicycling is limited in the United States. We explored the association between perceived social and built environment factors with total, transportation, and recreation bicycling in a sample of adult bicyclists in Austin, Texas, and Birmingham, Alabama. The objective of this study was to examine the relationship between perceived social and built environment factors and domain-specific bicycling in a sample of adult bicyclists.

**Methods:**

Adults aged 18 to 65 who rode a bicycle at least once in the past year completed an internet-based survey that was developed for this study to specifically assess correlates of bicycling; the study was conducted from October 2016 through January 2017. Perceived environmental factors assessed were residential density, traffic safety, destination, connectivity, safety from crime, aesthetics, and bicycle infrastructure. Multivariable logistic regression models were used to estimate the association of each perceived environmental factor (tertile 1, lowest; tertile 3, highest) with recreation-only and transportation bicycling. Effect modification of the relation between environmental factors and bicycling outcomes by sex was also examined.

**Results:**

The final analytic sample size was 801 participants. All environmental factors examined, including residential density, traffic safety, destinations, connectivity, aesthetics, bicycle infrastructure, and safety from crime showed significantly direct associations with transportation bicycling. Traffic safety, destinations, aesthetics, and bicycle infrastructure showed significant direct and inverse associations with recreation-only bicycling. Effect modification by sex was identified with residential density; a significant direct association with recreation-only bicycling was seen among women.

**Conclusion:**

These findings illustrate that bicycling for transportation is associated with different perceived environmental factors than is recreation-only bicycling, with some significant modification by sex. Comprehensive tools that assess the perceived environment for bikeability in the United States are warranted.

## Introduction

Bicycling is a physical activity behavior with known benefits to health and well-being (1,2). Bicyclists have a reduced risk of illness and death and improved cardiorespiratory fitness compared with both active and inactive nonbicyclists (2). Most of the US population is insufficiently active (3), but evidence suggests that bicycling is a way for people to meet physical activity guidelines for aerobic activity (4).

Evidence from around the world suggests that factors from multiple levels of the ecologic model (5) are associated with physical activity (6). Relative to the breadth of studies on physical activity in general, few studies have explored ecologic factors associated with bicycling, and those have shown mixed results (7–10). For example, perceived environmental correlates of bicycling in Belgium, where bicycling is common and supporting infrastructure is ubiquitous, differ from those in the United States (11), where the behavior and supporting infrastructure are much rarer (12). These findings suggest that local context plays an important role in these associations.

Additionally, often these studies have relied on assessment tools designed to identify perceived environmental factors related to walking (9,11,13–15). Given the unique nature of these two behaviors, walking and bicycling, it is likely that the factors influencing them vary (11). Evidence from the International Physical Activity and Environment Network project suggests that highly walkable environments may not support transportation bicycling (16).

Another limitation of past research is the lack of bicycling domain specificity — recreation and transportation. Limited research on recreation and transportation bicyclists has shown that different environmental factors are associated with bicycling by domain (17–19). Furthermore, sex has been identified as a potential effect modifier in the association between environmental factors and bicycling behavior by domain (20). The objective of this study was to explore the association between perceived social and built environment factors and domain-specific bicycling in a sample of adult bicyclists.

## Methods

### Study design

A cross-sectional study was conducted to assess ecological factors and their association with domain-specific bicycling among adults living in Travis County, Texas, and Jefferson County, Alabama. These sites were chosen to attain a wide range of perceived social and built environmental variability. Austin, Texas, located in Travis County, was awarded “gold status” for bicycling-friendliness in 2015 by the League of American Bicyclists (21). In contrast, Birmingham, Alabama, located in Jefferson County, has been identified as one of the worst US cities for bicycling (22).

An internet-based questionnaire was designed specifically for our study. A comprehensive review of the literature was conducted to identify existing instruments that have been used by others for examining correlates of bicycling behavior among adults. Furthermore, focus groups were conducted in Austin, Texas, and Birmingham, Alabama, to determine what factors bicyclists perceive as important for both adopting and maintaining bicycling behaviors. Focus groups were conducted for both transportation and recreation bicyclists, balanced in terms of length of riding, race/ethnicity, age, and sex. Information gleaned from the literature review and focus groups was used to create an initial questionnaire. This questionnaire was pretested in a small convenience sample of cyclists residing in the study areas, and a final version was developed by incorporating necessary revisions based on feedback.

Adult participants were recruited from October 2016 through January 2017 via the internet (Facebook, Reddit, Nextdoor), advertisements and by word of mouth. Recruitment was managed so that there was an equal number (±5%) of participants from the two study sites. People were eligible to participate if they reported living in the study area, were aged 18 to 65, and had ridden a bicycle at least once in the past year.

Data were collected and managed using Research Electronic Data Capture electronic data capture tools (REDCap) (23). REDCap is a secure, web-based application designed to support data capture for research studies. As incentive, participants were put into a drawing to win a $20 gift card. The University of Texas Health Institutional Review Board deemed this pilot study exempt from review.

### Study measures

Participants were identified as bicycling for transportation via the question: “In the past year, have you ridden a bicycle specifically for transportation (for example, to get to work, school, or other places)?” Those who answered yes were then asked: “Do you typically ride a bicycle for transportation at least once a month?” Similarly, participants were identified as bicycling for recreation via the question: “In the past year, have you ridden a bicycle specifically for recreation (for example, simply for fun, exercise, or competition)?” Those who answered yes were then asked: “Do you typically ride a bicycle for recreation at least once a month?” For the purposes of this analysis, participants were categorized into 1 of 3 groups (1). People who reported that they typically rode a bicycle for transportation at least once a month were categorized as transportation bicycling (2). Participants who reported they typically rode a bicycle for recreation at least once a month, and not identified as transportation bicycling, were categorized as recreation-only bicycling (3). Participants who reported that they did not typically ride a bicycle for recreation at least once a month, and not identified as transportation bicycling, were categorized as a nonbicycling. The sample size was not sufficient for a fourth category of transportation-only bicycling.

Perceived social and built environmental factors were assessed by using adapted questions from the Abbreviated Neighborhood Environment Walkability Scale (24), which included scales of residential density, destination, connectivity, safety from crime, and aesthetics (Appendix). For the purposes of this study, questions related to connectivity and aesthetics were modified to consider the routes the respondents took while riding a bicycle. Perceived social and built environment were further explored via a composite score of bicycle infrastructure by Handy and colleagues (25), which includes questions about bicycle lanes and street width. Finally, traffic safety was assessed via a composite score of perceived driver behavior based on an index by Handy and colleagues (25) that asked how drivers interact with bicyclists where respondents live.

Demographic variables used to describe the sample and as covariates were age (calculated from date of birth to date of questionnaire completion), sex, race/ethnicity, education, employment status, and household income; these were chosen a priori based on previous literature (10,11).

### Statistical analysis

Descriptive statistics were median and interquartile range (IQR) for continuous variables and proportion for categorical and nominal variables. We examined missing data patterns using Little's χ^2^ test for missing completely at random. We used multinomial logistic regression models to estimate the association of the perceived environmental variables with transportation and recreation-only bicycling. Nonbicycling was used as the referent for all models. Because of a lack of normality, we categorized perceived environmental variables into tertiles. We calculated the intracluster correlation coefficient (ICC) to assess clustering by study site; a significant ICC (*P* < .05) indicated the need to account for clustering (26).

For all models, we first examined each perceived environmental variable in independent bivariate multinomial logistic regression models. Perceived environmental variables that were significantly associated (*P* < .05) with one of the outcomes were then examined in a multivariable multinomial logistic regression model, adjusting for demographic covariates. Next, we conducted Wald tests for interactions between each perceived environmental variable and sex; a *P* value of <.15 indicated a significant interaction (26). If a significant interaction was identified, we examined results as a linear combination of coefficients by sex. We assessed collinearity via variance inflation factors (VIF); a VIF less than 10 indicated variable collinearity (27). Final regression estimates are reported as adjusted odds ratios (AOR) with 95% confidence intervals (CIs). Analyses were conducted using STATA version 13.1 (STATA Corporation).

## Results

A total of 998 people completed the survey. Missing data were missing completely at random (*P* > .05); thus, a complete case analysis was conducted. The final analytic sample was 801 (80.2%) participants. Fifty-three percent of the participants were from Travis County, Texas, and 47% were from Jefferson County, Alabama. The ICC was significant, so clustering by study site was accounted for in all models.

Overall, participants’ median age was 35.5 years; they were primarily male (55.4%), white (83.4%), college educated (highest level undergraduate or graduate, 77.7%), and employed (85.0%) (Table 1). Forty-eight percent reported a household income of $75,000 and above. Fourteen percent were categorized as nonbicycling, 34.0% were categorized as recreation-only bicycling, and 52% were categorized as transportation bicycling. Among those that rode a bicycle for transportation in the past year, 93% reported bicycling for recreation in the past year.

**Table 1:** Characteristics of Adult Bicyclists in Travis County, Texas, and Jefferson County, Alabama, October 2016–January 2017

Variable^a^	Total Sample, N = 801	Nonbicycling, n = 113	Recreation-Only Bicycling, n = 271	Transportation Bicycling, n = 417
**Demographic Variables**				
**Age in years, median (interquartile range)**	35.5 (16.9)	34.4 (15.2)	41.5 (17.6)	32.9 (14.6)
**Sex**				
Male	444 (55.4)	30 (26.5)	142 (52.4)	272 (65.2)
Female	357 (44.6)	83 (73.5)	129 (47.6)	145 (34.8)
**Race/ethnicity**				
White	668 (83.4)	93 (82.3)	231 (85.2)	344 (82.5)
Black	31 (3.9)	6 (5.3)	18 (6.6)	7 (1.7)
Hispanic	46 (5.7)	6 (5.3)	12 (4.4)	28 (6.7)
Other	56 (7.0)	8 (7.1)	10 (3.7)	38 (9.1)
**Education**				
Less than high school/ high school graduate or equivalent	41 (5.1)	9 (8.0)	13 (4.8)	19 (4.6)
Some college/associates degree	138 (17.2)	17 (15.0)	42 (15.5)	79 (18.9)
Undergraduate degree	353 (44.1)	47 (41.6)	121 (44.7)	185 (44.4)
Graduate degree	269 (33.6)	40 (35.4)	95 (35.1)	134 (32.1)
**Employed part-time or full-time**				
Yes	681 (85.0)	93 (82.3)	237 (87.4)	351 (84.2)
No	120 (15.0)	20 (17.7)	34 (12.6)	66 (15.8)
**Annual household income, $**				
< 30,000	132 (16.5)	16 (14.2)	20 (7.4)	96 (23.0)
30,000 to <75,000	282 (35.2)	37 (32.7)	87 (32.1)	158 (37.9)
≥75,000	387 (48.3)	60 (53.1)	164 (60.5)	163 (39.1)
**Perceived Environment Variables**				
**Residential density**				
Tertile 1	330 (41.2)	65 (57.5)	148 (54.6)	117 (28.1)
Tertile 2	204 (25.5)	19 (16.8)	60 (22.1)	125 (30.0)
Tertile 3	267 (33.3)	29 (25.7)	63 (23.3)	175 (42.0)
**Destination**				
Tertile 1	298 (37.2)	55 (48.7)	142 (52.4)	101 (24.2)
Tertile 2	361 (45.1)	49 (43.4)	94 (34.7)	218 (52.3)
Tertile 3	142 (17.7)	9 (8.0)	35 (13.0)	98 (23.5)
**Connectivity**				
Tertile 1	299 (37.3)	48 (42.5)	116 (42.8)	135 (32.4)
Tertile 2	280 (35.0)	42 (37.2)	101 (37.3)	137 (32.9)
Tertile 3	222 (27.7)	23 (20.4)	54 (19.9)	145 (34.8)
**Safety from crime**				
Tertile 1	233 (29.1)	45 (39.8)	72 (26.6)	116 (27.8)
Tertile 2	221 (27.6)	22 (19.5)	60 (22.1)	139 (33.3)
Tertile 3	347 (43.3)	46 (40.7)	139 (51.3)	162 (38.9)
**Aesthetics**				
Tertile 1	453 (56.6)	74 (65.5)	145 (53.5)	234 (56.1)
Tertile 2	103 (12.9)	11 (9.7)	36 (13.3)	56 (13.4)
Tertile 3	245 (30.6)	28 (24.8)	90 (33.2)	127 (30.5)
**Bicycle infrastructure**				
Tertile 1	300 (37.5)	52 (46.0)	122 (45.0)	126 (30.2)
Tertile 2	241 (30.1)	32 (28.3)	75 (27.7)	134 (32.1)
Tertile 3	260 (32.5)	29 (25.7)	74 (27.3)	157 (37.7)
**Traffic safety**				
Tertile 1	277 (34.6)	42 (37.2)	104 (38.4)	131 (31.4)
Tertile 2	348 (43.5)	43 (38.1)	116 (42.8)	189 (45.3)
Tertile 3	176 (22.0)	28 (24.8)	51 (18.8)	97 (23.3)

a Values are n (%) unless otherwise noted.

In the bivariate multinomial logistic regression (Table 2), as compared with nonbicycling, at least one level of destination and safety from crime was significantly associated with both recreation-only and transportation bicycling. Traffic safety, residential density, connectivity, and infrastructure were significantly associated with transportation bicycling. Aesthetics was significantly associated with recreation-only bicycling.

**Table 2:** Bivariate Multinomial Logistic Regression Models, Adult Bicyclists (N = 801) in Travis County, Texas, and Jefferson County, Alabama, October 2016–January 2017

Perceived Environment Variable^a^	Recreation-Only Bicycling^b^, OR (95% CI)	Transportation Bicycling^b^, OR (95% CI)
**Residential density**		
Tertile 2	1.39 (0.81–2.38)	3.65 (2.33–5.73)^c^
Tertile 3	0.95 (0.69–1.31)	3.35 (1.96–5.73)^c^
**Traffic safety**		
Tertile 2	1.09 (0.85–1.39)	1.41 (1.25–1.58)^c^
Tertile 3	0.74 (0.42–1.29)	1.11 (0.44–2.83)
**Destination**		
Tertile 2	0.74 (0.62–0.89)^c^	2.42 (2.31–2.54)^c^
Tertile 3	1.51 (1.11–2.04)^c^	5.93 (2.76–12.76)^c^
**Connectivity**		
Tertile 2	1.00 (0.90–1.10)	1.16 (1.06–1.27)^c^
Tertile 3	0.97 (0.89–1.06)	2.24 (2.10–2.39)^c^
**Aesthetics**		
Tertile 2	1.67 (0.70–3.96)	1.61 (0.52–5.01)
Tertile 3	1.64 (1.33–2.02)^c^	1.43 (0.85–2.41)
**Infrastructure**		
Tertile 2	1.00 (0.93–1.08)	1.73 (0.93–1.08)
Tertile 3	1.09 (0.64–1.84)	2.23 (1.29–3.88)^c^
**Safety from crime**		
Tertile 2	1.70 (0.72–4.05)	2.45 (1.70–3.53)^c^
Tertile 3	1.89 (1.22–2.92)^c^	1.37 (0.53–3.49)

Abbreviations: CI, confidence interval; OR, odds ratio.

a Referent is tertile 1 for all models. Tertile 1 represents the lowest scores for that perceived environmental variable, and tertile 3 represents the highest scores for that variable.

b As compared to nonbicycling.

c *P* < .05.

The multivariable multinomial logistic regression models, adjusted for demographic variables, for recreation-only and transportation bicycling are reported (Table 3). The VIF indicated that collinearity could be discarded as a concern. The second tertile of perceived traffic safety was significantly associated with recreation-only (AOR = 1.12; 95% CI, 1.05–1.21) and transportation bicycling (AOR = 1.44; 95% CI, 1.37–1.50), but not the third tertile. Higher access to destinations by bicycle was significantly associated with both recreation-only and transportation bicycling; tertile 2 was associated with lower odds (AOR=0.73;95% CI 0.66–0.81) and tertile 3 was associated with higher odds of recreation-only bicycling (AOR = 1.42; 95% CI, 1.07–1.89), whereas both tertiles were associated with higher odds of transportation bicycling (tertile 2, AOR = 2.31; 95% CI, 1.92–2.78; tertile 3, AOR = 6.80; 95% CI, 3.18–14.53). The highest level of connectivity was significantly associated with transportation bicycling (AOR = 2.11; 95% CI, 1.84–2.41). The highest level of aesthetically pleasing routes was significantly associated with both recreation-only (AOR = 1.57; 95% CI, 1.41–1.74) and transportation bicycling (AOR = 1.48; 95% CI, 1.10–1.82). Bicycle infrastructure was associated with recreation-only bicycling at tertile 2 (AOR = 1.03; 95% CI, 1.03–1.04), but not at tertile 3 (AOR = 1.15; 95% CI, 0.67–1.98). The highest level of bicycle infrastructure was significantly associated with transportation bicycling (AOR = 3.45; 95% CI, 1.43–4.18). A higher perceived safety from crime was significantly associated with a higher odds of transportation bicycling (tertile 2, AOR = 2.38; 95% CI, 1.34–4.23), but not at the highest level.

**Table 3:** Multivariable Multinomial Logistic Regression Models, Adult Bicyclists (N = 801) in Travis County, Texas, and Jefferson County, Alabama, October 2016–January 2017

Perceived Environment Variable^a^	Recreation-Only Bicycling^b^, AOR (95% CI)	Transportation Bicycling^b^, AOR (95% CI)
**Residential density^c^**		
Men		
Tertile 2	2.13 (0.81–5.60)	4.20 (1.85–9.51)^d^
Tertile 3	1.11 (0.43–2.89)	2.48 (1.04–5.95)^d^
Women		
Tertile 2	1.06 (0.66–1.72)	3.62 (3.21–4.07)^d^
Tertile 3	1.06 (1.00–1.12)^d^	3.14 (2.16–4.56)^d^
**Traffic safety**		
Tertile 2	1.12 (1.05–1.21)^d^	1.44 (1.37–1.50)^d^
Tertile 3	0.73 (0.45–1.19)	1.10 (0.45–2.70)
**Destination**		
Tertile 2	0.73 (0.66–0.81)^d^	2.31 (1.92–2.78)^d^
Tertile 3	1.42 (1.07–1.89)^d^	6.80 (3.18–14.53)^d^
**Connectivity**		
Tertile 2	1.02 (0.81–1.28)	1.05 (0.85–1.29)
Tertile 3	1.07 (0.90–1.27)	2.11 (1.84–2.41)^d^
**Aesthetics**		
Tertile 2	1.48 (0.48–4.58)	1.54 (0.43–5.57)
Tertile 3	1.57 (1.41–1.74)^d^	1.48 (1.20–1.82)^d^
**Infrastructure**		
Tertile 2	1.03 (1.03–1.04)^d^	1.61 (0.84–3.10)
Tertile 3	1.15 (0.67–1.98)	3.45 (1.43–4.18)^d^
**Safety from crime**		
Tertile 2	1.47 (0.56–3.86)	2.38 (1.34–4.23)^d^
Tertile 3	1.52 (0.85–2.71)	1.38 (0.55–3.50)

Abbreviations: AOR, adjusted odds ratio; CI, confidence interval.

a Models were adjusted for age, education, income, employment, and race, controlling either for sex or presented as a linear combination by sex. Referent is tertile 1 for all models. Tertile 1 represents the lowest scores for that perceived environmental variable, and tertile 3 represents the highest scores for that variable.

b As compared to nonbicycling.

c A significant interaction between this perceived built environment variable and sex was observed (Wald test *P* < 0.15). The association of this perceived built environment variable with recreation bicycling are presented as a linear combination of coefficients by sex.

d *P* < .05.

The effects of residential density on the studied bicycling behavior outcomes were modified by sex. As residential density increased, men had significantly higher odds of transportation bicycling (tertile 2, AOR = 4.20; 95% CI, 1.85–9.51; tertile 3, AOR = 2.48; 95% CI, 1.04–5.95). For women, as residential density increased, there were significant higher odds of transportation bicycling (tertile 2, AOR = 3.62; 95% CI, 3.21–4.07; tertile 3, AOR = 3.14; 95% CI, 2.16–4.56); furthermore, the highest tier of residential density was associated with recreation-only bicycling (AOR = 1.06; 95% CI, 1.00–1.12).

## Discussion

Our study examined perceived social and built environment factors and their association with recreation-only and transportation bicycling among a sample of bicyclists residing in Jefferson County, Alabama, and Travis County, Texas. The perceived environmental factors found to be significantly associated with bicycling differed by domain. These associations were further examined by sex; significant interactions between sex and perceived environmental variables were identified. The patterns of interaction differed by bicycling domain.

Overall, the perceived environment appeared to be associated more with transportation bicycling, both in terms of strength of the associations and in the number of perceived environment factors significantly associated with the behavior. These findings illustrate that the perceived environment correlates of transportation and recreation-only bicycling differ. Our findings stress that bicycling is not a homogeneous behavior. It is important to consider recreation and transportation bicycling as independent activities, motivated and influenced by different factors.

Differences by bicycling domain were also observed with respect to residential density when examining the potential effect modification of sex on the association between perceived environment variables and bicycling. In our study, residential density was measured as type of housing (ie, single family, apartments) in the participant’s neighborhood. Residential density was significantly associated with transportation bicycling for both men and women. In addition, the highest level of residential density had a significant association with recreation-only bicycling among women. Previous research has reported that men are generally more physically active than women and are more likely to be bicyclists (15). The explanations of these differing physical activity behaviors by sex are surely complex and due to multiple factors, but the perceived environment appears to be influential (20). Future research should continue to explore these concepts and how they might differentially affect bicycling among men and women.

Perceived bicycling infrastructure was associated with both recreation-only and transportation bicycling. This builds upon previous evidence of an association between perceived bicycling infrastructure and bicycling in the United States (28). Furthermore, a moderate level of traffic safety was identified as being important for both recreation-only and transportation bicycling, but not at the highest level. Similarly, transportation bicycling was associated with safety from crime at the mid level, but not at the highest level. How these different perceptions of the social and built environment interact and influence bicycling warrants further study.

One of the few other studies to examine the association between environmental perceptions and recreation-only and transportation bicycling was conducted by Heesch, Giles-Corti, and Turrell (10) in a sample of adults aged 40 to 65 residing in Brisbane, Australia (10). The methods used in our study were in part modeled after that study, and the findings seen in our study are largely comparable to what was observed in their sample, in that the perceived environment seemed overall more significantly associated with transportation bicycling than with recreation-only bicycling. However, the patterns of association by domain differed by study. There were notable differences between the ages of the participants and operationalization of the perceived environmental concepts. Despite these differences, findings indicate that country, and even region within a country, is an important consideration for any inquiry into how perceptions of the environment influence physical activity.

Taken together, many of the Neighborhood Environment Walkability Scale (NEWS) variables were associated with bicycling, but the patterns of association were more complex than previously reported. Sallis and colleagues (15) used NEWS to measure associations between perceived environment variables and bicycling frequency in 2 US cities — Baltimore, Maryland, and Seattle, Washington. They found that only one of the NEWS variables, destination, was significantly associated with bicycling frequency (15). One important difference between our work and their study is that theirs did not examine bicycling domains. A large, multicountry study by Kerr and colleagues (16) used NEWS to assess the association between perceived environmental variables and transportation bicycling. That study reported that many perceived built environment factors were significantly associated with engaging in any bicycling over the past week, but that only traffic safety and crime safety were associated with minutes per week of transportation bicycling (16). In comparison, many NEWS-related variables were associated with transportation bicycling in our study.

A notable difference between these previous studies and our current work is that we adapted NEWS to be specifically applicable to bicycling rather than to the more general walkability measures used by Kerr and Sallis (15,16). This may partly explain the differing findings. In all, the results from these studies suggest that measures designed to capture walkability (eg, NEWS) may not be the best suited for understanding the influences of the perceived built environment on bicycling. Future research should focus on developing better measures to capture the construct of bikeability in neighborhoods and routes, considering bicycling domains.

This study had many strengths. We collected bicycling behavior and perceived environment data from a sample of adults from 2 environmentally diverse counties in the United States, thus maximizing the variability of our exposure of interest. Both neighborhood- and route-based measures of the perceived social and built environment were used. This represents an innovation for exploring the potential drivers of bicycling, independent of walking, active travel, or other physical activity. The purposeful identification of both transportation and recreation bicycling behaviors, and the analysis of each independently is also a strength, especially considering the dearth of work examining the factors influencing different bicycling domains.

This study also had limitations. The convenience sample precludes generalizability of findings; caution is necessary in interpreting results. Although using a random sample would have been ideal, there were several challenges associated with this. The lack of a census of adults that had bicycled at least once in the past year in Travis and Jefferson counties made random sampling expensive and logistically complicated. Alternatively, if we had used a random sample of adults regardless of whether they had bicycled or not over the past year, we would be challenged by the low prevalence of transportation bicycling in the United States (approximately 8%) (12), thus requiring an excessively large sample size. Although the sample size overall was robust, small cell sizes influenced the stability of some estimates. The use of self-report supposes inherent risks of information bias (29). Not all items used to measure the perceived environment have been validated for bicycling. Finally, because of the cross-sectional design of the study, causality could not be determined.

Our findings have several implications for practice. First, it is imperative that practitioners target their interventions to a particular domain, while at the same time considering how a particular change may inadvertently affect other bicycling populations outside of their intended target. In addition, it is important to recognize that perceptions of the built environment may differ from what is objectively measured (28). Interventions that strive to increase bicycling behavior should consider not only aspects of the environment that influence bicycling, such as bike lanes, but also how they are perceived by the population. Furthermore, the influence of sex on these perceptions is worth consideration when exploring potential built environment changes.

Among our sample of adult bicyclists, it was evident that perceptions of the social and built environment differed by bicycling domain, recreation and transportation. For some perceived environmental factors, these perceptions further differed for men and women. Future research should consider domain when investigating potential correlates of bicycling, as well as how these factors differentially influence men and women. Future research should aim to have more diverse and representative samples, including representation from racial/ethnic minorities and low-income populations. New, better measures of the perceived environment as it relates to domain-specific bicycling are warranted. For a community to be truly “activity promoting,” urban and transportation planning should aim to accommodate both walking and bicycling as well as other physical activities.

## Appendix Questionnaire Items Assessing the Social and Built Environment, Titled by the Variable They Are Measuring, With Information on Scoring

**Table:** Traffic Safety^a^

What are drivers like where you ride?	Strongly disagree (1)	Some-what disagree (2)	Some-what agree (3)	Strongly agree (4)
TS1. Most drivers seem oblivious to bicyclists (reverse scored)				
TS2. Most drivers yield to bicyclists				
TS3. Most drivers watch for bicyclists at intersections				
TS4. Most people do not drive faster than the speed limit				

^a^ Traffic safety scoring: TS = (TS1 + TS2 +TS3 + TS4)/4.

**Table:** Residential Density

How common are the following housing types in the neighborhood where you live?^a^	None (1)	A few (2)	Some (3)	Most (4)	All (5)
A1. Detached single-family residences					
A2. Townhouses or row houses					
A3. Apartment or condos 1-3 stories					
A4. Apartments or condos 4-6 stories					

^a^ Residential scoring: A = A1 + (12 × A2) + (10 × A3) + (25 × A4).

**Table:** Destination^a^

These questions are about where you can go in the neighborhood where you live. Think of biking distance as within a 10-15 minute bike ride from your home.	Strongly disagree (1)	Somewhat disagree (2)	Somewhat agree (3)	Strongly agree (4)
C1. Stores are within easy biking distance of my home				
C2. There are many places to go within easy biking distance of my home				
C3. It is easy to bike to a transit stop (bus, train) from my home				
C4. Parking my car is difficult in local shopping areas (reverse scored)				

^a^ Destination scoring: C = (C1 + C2 + C3 + C4)/4.

**Table:** Connectivity^a^

Please indicate the answer that best applies to the roads that you ride on.	Strongly disagree (1)	Somewhat disagree (2)	Somewhat agree (3)	Strongly agree (4)
D1. The distance between intersections in my neighborhood is usually short (100 yards or less; the length of a football field or less)				
D2. There are many alternative routes for getting from place to place (I don’t have to go the same way every time)				
D3. The streets where I ride do not have many cul-de-sacs (dead-end streets)				
D4. There are major barriers to biking in my local area that make it hard to get from place to place (for example, freeways, railway lines, rivers) (reverse scored)				

^a^ Connectivity scoring: D = (D1 + D2 + D3 + D4)/4.

**Table:** Safety From Crime^a^

These questions are about crime in the neighborhood where you live.	Strongly disagree (1)	Somewhat disagree (2)	Somewhat agree (3)	Strongly agree (4)
E1. There is a high crime rate in my neighborhood (reverse scored)				
E2. The crime rate in my neighborhood makes it unsafe to go on bike rides during the day (reverse scored)				
E3. The crime rate in my neighborhood makes it unsafe to go on bike rides at night (reverse scored)				

^a^ Safety from crime scoring: E = (E1 + E2 + E3)/3.

**Table:** Aesthetics^a^

The following questions are about how your regular bike routes look.	Strongly disagree (1)	Somewhat disagree (2)	Somewhat agree (3)	Strongly agree (4)
F1. There are trees along the streets on my bike routes				
F2. There are many interesting things to look at on my bike routes				
F3. There are many attractive natural sights on my bike route (such as landscaping, views)				
F4. There are attractive buildings/homes on my bike route				

^a^ Aesthetics scoring: F = (F1 + F2 + F3 + F4) / 4.

**Table:** Bicycle Infrastructure^a^

These next questions are about how your city is designed for biking.	Strongly disagree (1)	Somewhat disagree (2)	Somewhat agree (3)	Strongly agree (4)
I1. Major streets have bike lanes				
I2. Streets without bike lanes are generally wide enough to bike on				
I3. Store and other destinations have bike racks				
I4. Streets and bike paths are well lighted				
I5. The city has a network of off-street bike paths				
I6. Bike lanes are free of obstacles				
I7. The bike route network has big gaps (reverse scored)				
I8. The area is too hilly for easy biking (reverse scored)				

^a^ Bicycle infrastructure scoring: I = (I1 + I2 + I3 +I4 + I5 +I6 + I7 + I8)/8.
